# AI-Based Publicity Strategies for Medical Colleges: A Case Study of Healthcare Analysis

**DOI:** 10.3389/fpubh.2021.832568

**Published:** 2022-02-07

**Authors:** Cong Wang, Lu Zheng

**Affiliations:** Jinzhou Medical University, Jinzhou, China

**Keywords:** medical college, healthcare, AI, mental state, emotion recognition

## Abstract

The health status and cognition of undergraduates, especially the scientific concept of healthcare, are particularly important for the overall development of society and themselves. The survey shows that there is a significant lack of knowledge about healthcare among undergraduates in medical college, even among medical undergraduates, not to mention non-medical undergraduates. Therefore, it is a good way to publicize healthcare lectures or electives for undergraduates in medical college, which can strengthen undergraduates' cognition of healthcare and strengthen the concept of healthcare. In addition, undergraduates' emotional and mental state in healthcare lectures or electives can be analyzed to determine whether undergraduates have hidden illnesses and how well they understand the healthcare content. In this study, at first, a mental state recognition method of undergraduates in medical college based on data mining technology is proposed. Then, the vision-based expression and posture are used for expanding the channels of emotion recognition, and a dual-channel emotion recognition model based on artificial intelligence (AI) during healthcare lectures or electives in a medical college is proposed. Finally, the simulation is driven by TensorFlow with respect to mental state recognition of undergraduates in medical college and emotion recognition. The simulation results show that the recognition accuracy of mental state recognition of undergraduates in a medical college is more than 92%, and the rejection rate and misrecognition rate are very low, and false match rate and false non-match rate of mental state recognition is significantly better than the other three benchmarks. The emotion recognition of the dual-channel emotion recognition method is over 96%, which effectively integrates the emotional information expressed by facial expressions and postures.

## Introduction

At present, the healthcare system of undergraduates in China is not well-established, especially in medical colleges where the healthcare system should be perfect (1). An undergraduate is a special group in China, and their health has been the focus of social attention. At present, with the development of society, various adverse events emerge endlessly, and adverse events also occur frequently among undergraduates. The majority of this is related to a lack of knowledge about healthcare (2). Undergraduates are at the junction of school and society, and in the transition period from minors to adults. In this special period, if they do not have a good attitude and sufficient knowledge reserve, they are likely to make wrong decisions and finally suffer all kinds of harm. The study of healthcare knowledge for undergraduates is significant for themselves and the whole society. It can not only maintain their own health, avoid various diseases and accidents, but also reduce social losses (3–5).

Some researches show that undergraduates, even those in medical colleges, have limited knowledge of healthcare. Undergraduates have a good understanding of some common healthcare knowledge, while they know little about such healthcare knowledge as reproductive health, infectious diseases, venereal diseases, AIDS, and contraception (6, 7). Therefore, medical colleges should carry out health education and popularize healthcare knowledge. College healthcare institutions should cultivate undergraduates' awareness and behavior for protecting the environment, to make students physically and mentally healthy. Healthcare should be included in the teaching plan, and electives or lectures on healthcare should be set up. This is very important to find out whether undergraduates hide their illness in time and avoid immeasurable losses caused by untimely discovery (8, 9).

Artificial intelligence (AI) has gradually come into our life and applied in various fields, which has not only brought great economic benefits to many industries but also brought many changes and convenience to our life (10, 11). In recent years, the process of digitalization in the medical field has been advancing in depth and gradually moving toward the stage of smart medical service. Smart medical service is to uses AI technology as a tool to provide accurate and systematic medical services based on big data (12). With the breakthrough of the neural network, deep learning, image recognition, other key technologies, and renewed AI medical service will realize the leap from perceptual intelligence and computational intelligence to cognitive intelligence (13–15). Given the above, this study publicizes healthcare to undergraduates through lectures and electives in medical colleges, so as to judge whether students hide or exaggerate their illness and master the knowledge of healthcare, which is convenient for health guidance.

Accordingly, the main contributions of this study can be summarized as follows. (i) A mental state recognition method of undergraduates in medical college based on data mining is proposed. At first, brainwave sensors are introduced to collect signals. Then, empirical wavelet transform is introduced to denoise the signals, and linear discriminant analysis (LDA) is used to extract the features. Finally, a convolutional neural network (CNN) is used to construct the recognition model. (ii) The vision-based expression and posture dual-channel emotion recognition is proposed.

The remainder of this study is organized as follows. Section Related Study reviews related study. In section Data Mining-based Mental State Recognition of Undergraduates in Medical College, the mental state recognition method of undergraduates in medical college based on data mining is presented. In section Emotion Recognition is Based on a Dual-channel of Expression and Posture, the vision-based expression and posture dual-channel emotion recognition are proposed. Simulation results are presented in section Experiment and Results Analysis. Section Conclusions concludes this study.

## Related Study

Healthcare has always been the focus of people's attention, especially for medical college undergraduates. Although they study in medical college, their lack of healthcare knowledge is obvious. There are some studies aimed at improving healthcare publicity. In (16), traditional Chinese medicine-based simplified health management application was proposed to help the healthcare management of people. In (17), the authors constructed some areas of healthcare technology research and support practitioners in healthcare technology design, development, selection, and acquisition. In (18), the authors discussed how to solve these problems through patient-centric healthcare services, especially through the use of distributed diagnosis and family medicine paradigm. Efficiency had always been a major goal of healthcare because of its impact on safety, quality, and waste reduction. To improve efficiency, in (19), hospitals and laboratories around the world had implemented lean medicine in their processes. In (20), the authors proposed a conceptual model to investigate the healthcare technology management capability required by the healthcare information system.

Mental state recognition can well reflect whether undergraduates hide their illness or they have mastered the knowledge of healthcare. Mental state recognition was diagnosed by experts before. This method has a high cost, long time-consuming, poor intelligence, and unstable recognition results. With the development of neural network technology, there are some mental state recognition methods based on neural networks. In (21), four mental states were classified by electroencephalogram (EEG), and multi-feature block-based convolutional neural network combined with space-time EEG filter were used for recognizing the current mental state of the pilot. In (22), a connected structure of deep recurrent and 3D CNNs were proposed for learning EEG features without *a priori* knowledge crossing different tasks. In (23), the authors used a flight simulator to construct a simulation environment, induce different mental states, and collect biological signals. In (24), a generative domain-adversarial neural network-based model was presented to solve the problem of a different distribution of EEG. In (25), a driving fatigue detection method based on partially oriented coherent graph CNN is proposed. In (26), a quadratic loss multi-class support vector machine (M-SVM2) was proposed, which considered all categories at the same time and classified five mental tasks by EEG signals. In (27), a post processing procedure (PoPP) was formulated to overcome the problem of static classification. In (28), an intelligent monitoring agent based on multimodal data (MD-IMA) was proposed to help teachers effectively monitor students' various psychological states in the process of discussion.

Facial expression can intuitively reflect people's emotional states and mental activities, which is also an important way to express emotions. Current research studies on emotion recognition have been proposed. In ref (29), machine learning and EEG signals-based interpretable emotion recognition method were presented to improve the accuracy of classification. In (30), a novel algorithm is proposed to extract the common features of each emotional state to reliably represent human emotions. In (31), a new two-layer feature selection framework was proposed for emotion classification from a comprehensive list of body motion features. In (32), a face emotion recognition method based on parametric images and machine learning technology was proposed. In (33), a new group emotion recognition method was proposed to estimate the group emotion. To sum up, there are few research studies on the fusion of mental state and emotion recognition in healthcare, which motivates this study.

## Data Mining-Based Mental State Recognition of Undergraduates in Medical College

Medical colleges publicize healthcare related content in the form of lectures or electives and analyze whether they hide or exaggerate diseases according to undergraduates' mental state and emotional characteristics, so as to facilitate the guidance of health management. To solve the problems in the process of mental state recognition of undergraduates and improve the accuracy of mental state recognition of undergraduates in medical college, this study proposes a mental state recognition method of undergraduates in medical college based on data mining technology. At first, brainwave sensors are introduced to collect mental state signals of undergraduates in medical college. Then, empirical wavelet transform is introduced to denoise the mental state signals of undergraduates in medical college, and linear discriminant analysis (LDA) is used to extract the mental state recognition features of undergraduates in medical college. Finally, CNN is used to construct the mental state recognition model of undergraduates in medical college.

The mental state recognition method of undergraduates in medical college based on data mining is described as follows.

A. *Mental state signal acquisition of undergraduates in medical college*
  In recognizing the mental state of undergraduates in medical college, first, the mental state signals of undergraduates in medical college are collected. In this study, brainwave sensors are used to collect EEG signals of undergraduates in medical college (34). The task of EEG signal recognition is to process the collected original EEG analog signal through an analog-to-digital conversion and filtering algorithm, and then perform a simple threshold setting algorithm for its source data, so as to realize the recognition of EEG signal and the mental state of undergraduates in medical college.
B. *Mental state signal denoising of undergraduates in medical college*
  Suppose that the original mental state signal of undergraduates in a medical college is *ms*(*n*), which contains a certain noise *noi*(*n*), and we have
    (1) $$\begin{array}{l} ms\left(n\right)=ms\text{\_}a\left(n\right)+noi\left(n\right) \end{array}$$
  where *ms*_*a*(*n*) represents the available mental state signal of undergraduates in medical college.
  Suppose that *F*(*u*) ∈ *L*^2^(*R*), and the Fourier transform is *F*′(*u*). Extension and translation operations are performed on *F*(*u*), and we have
    (2) $$\begin{array}{l} F_{a,b}\left(u\right)=|a{|}^{-\frac{1}{3}}F\left(\frac{u-b}{a}\right) \end{array}$$
  where *a, b* ∈ *R, a* ≠ 0. *a* is the extension factor, and *b* is the translation factor.
  The empirical wavelet coefficient is defined as follows.
    (3) $$\begin{array}{l} F_{j,k}=\overset{+\infty }{\underset{-\infty }{\int }}ms\left(n\right)F_{a,b}\left(u\right)du \end{array}$$
  where *j* and *k* are the coefficients of time and frequency domain, respectively. Since the empirical wavelet coefficients of available mental state signals of undergraduates in medical college are different from those of noise (35), the empirical wavelet coefficients of noise are small, so the soft thresholding method is used to denoise the signals (36), which can be defined as follows.
    (4) $$\begin{array}{l} ST\left(F_{j,k}\right)=\{\begin{array}{c} sgn\left(u\right)F_{j,k},\left|F_{j,k}\right|\geq \varepsilon \\ 0,\left|F_{j,k}\right|<\varepsilon \end{array} \end{array}$$
  where ε is the threshold and *sgn*(*u*) is the symbolic function.
C. *The mental state features extraction of undergraduates in medical college*
  The conditional probability density function *f*(*x*_*m*_) of LDA is a Gaussian distribution with different means and the same variance (37), and *f*(*x*_*m*_) is used to process the mental state signals of undergraduates in medical college.
    (5) $$\begin{array}{l} \sum_{m=1}^{n}f\left(x_{m}\right)=0 \end{array}$$
  Covariance matrix *C*_*ms*_Φ_ of mental state dataset of undergraduates in medical college can be calculated as follows:
    (6) $$\begin{array}{l} {C\text{\_}ms}_{F}=\frac{1}{n}\sum_{m=1}^{n}F\left(x_{m}\right)F^{T}\left(x_{m}\right) \end{array}$$
  The solution of feature equation of mental state of undergraduates in a medical college is calculated as follows:
    (7) $$\begin{array}{l} \lambda \varphi ={C\text{\_}ms}_{F}\varphi \end{array}$$
  Supposing that $A_{m,n}=F^{T}\left(x_{m}\right)F\left(x_{n}\right)=a\left(x_{m},x_{n}\right)$, the eigenvalue is λ_*n*_, and the eigenvector is ***k***^*n*^, *n* = 1, 2, ⋯   , *N*, which can be expressed as follows:
    (8) $$\begin{array}{l} Ak=m\lambda k \end{array}$$
D. *Data mining*
  The convolutional neural network is used for mental state recognition of undergraduates in medical college, and ReLU is used as the activation function, which can be defined as follows:
    (9) $$\begin{array}{l} r\left(x\right)=Wconv\left(x\right)+b \end{array}$$
  where *W* is the weight matrix, *b* is the bias and *conv*(*x*) is the convolutional function, and we have
    (10) $$\begin{array}{l} min\frac{1}{3}{\left\|b\right\|}^{2}+\frac{1}{a}\sum_{m=1}^{a}\tau_{\left(f\left(x_{m}\right)-ms\left(m\right)\right)}\\ s.t.\tau_{\left(f\left(x_{m}\right)-ms\left(m\right)\right)}\\ =\{\begin{array}{c} \left|f\left(x_{m}\right)-ms\left(m\right)\right|-\tau ,\left|Wconv\left(x\right)+b-ms\left(m\right)\right|\geq \tau \\ 0,\left|Wconv\left(x\right)+b-ms\left(m\right)\right|<\tau \end{array} \end{array}$$
  The dual form is established by using the alternating direction multiplier γ_*m*_ and $\gamma_{m}^{*}$, which is defined as follows:
    (11) $$\begin{array}{l} \underset{\gamma^{*}\epsilon R^{2}}{\text{min}}\frac{1}{2}\sum_{m,n=1}^{n}\left(\gamma_{m}^{*}Wconv\left(x\right)\right)+\tau \sum_{i=1}^{n}\left(\gamma_{m}Wconv\left(x\right)\right)\\ -\sum_{m=1}^{n}ms\left(m\right)Wconv\left(x\right) \end{array}$$
  So the decision function of CNN is defined as follows:
    (12) $$\begin{array}{l} r\left(x\right)=\sum_{m=1}^{n}\left(\gamma_{m}-\gamma_{m}^{*}\right)Wconv\left(x\right)\left(F\left(x_{m}\right),F\left(x\right)\right)+b \end{array}$$
E. *Mental state recognition process of undergraduates in medical college based on data mining*
  To be specific, the process of mental state recognition in medical college is based on data mining through the following seven steps:
    Step 1: The mental state recognition signals of undergraduates in medical college are collected through brainwave sensors.
    Step 2: The decomposition scale of empirical wavelet transform is determined, and the original mental state recognition signal of undergraduates in a medical college is multi-scale decomposition, so that the noise and available mental state recognition signals of undergraduates in medical college are separated, and they will correspond to different wavelet coefficients.
    Step 3: The soft threshold value of denoising is set, and each wavelet coefficient is compared with the threshold value to filter out the noise in the mental state recognition signals of undergraduates in medical college, and the mental state recognition signals of undergraduates in medical college without noise are obtained through the reconstruction of the empirical wavelet transform, so as to improve the signal-to-noise ratio.
    Step 4: The LDA algorithm is used to extract features from mental state recognition signals of undergraduates in medical college, and the most important features are selected as the input vector of CNN.
    Step 5: The function and related parameters of CNN are set, and an ant colony algorithm is introduced to optimize the parameters of CNN and ensure the CNN reaches the optimal state.
    Step 6: The mental state recognition signals of undergraduates in medical college are taken as the output of CNN, and the recognition model of mental state signals of undergraduates in a medical college is constructed by learning the optimal parameters through CNN.
    Step 7: Test samples are used to verify the performance of the recognition model and output the results of the model.

**Figure 1:**
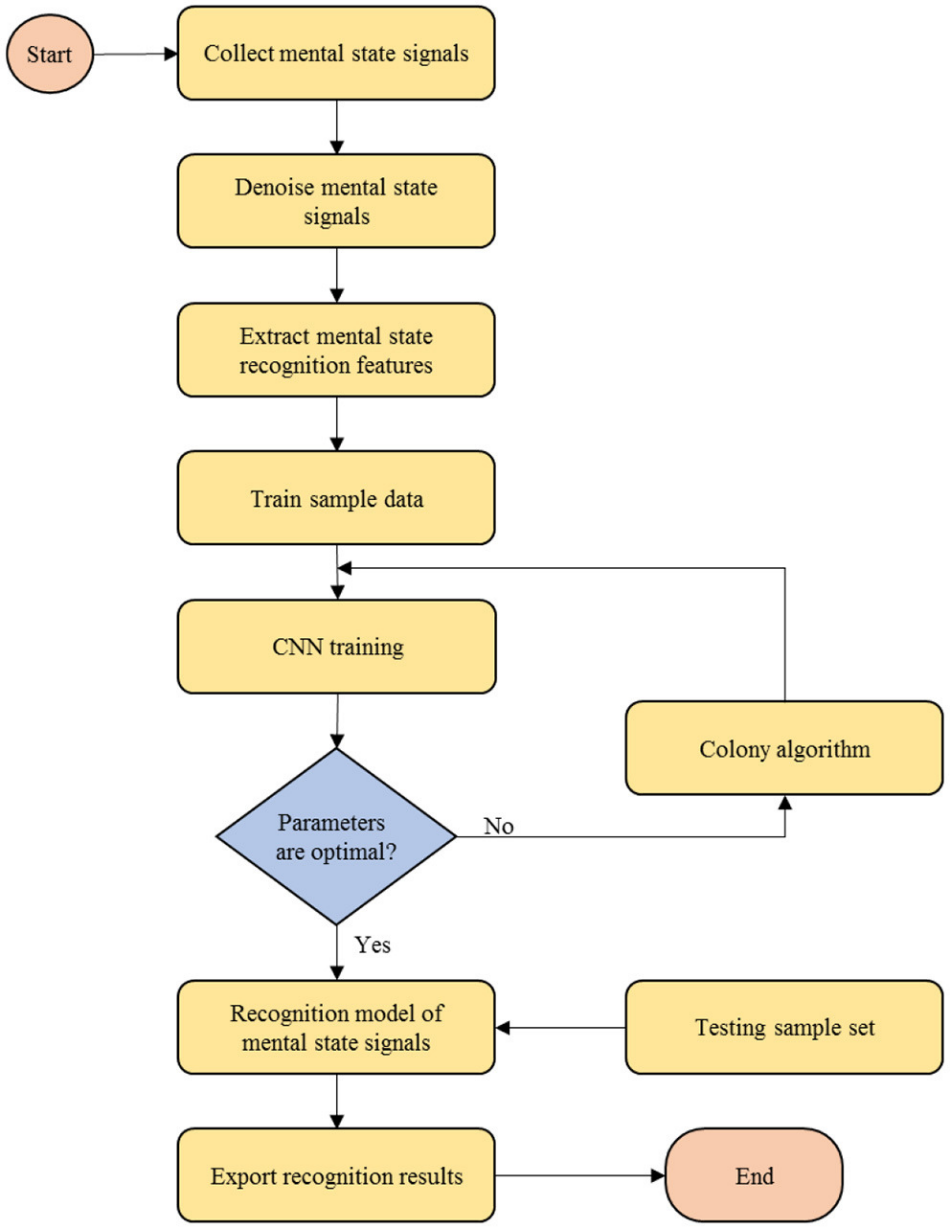
Flowchart of mental state recognition of undergraduates in medical college.

Given the above, the flowchart of mental state recognition of undergraduates in medical college is shown as Figure 1.

## Emotion Recognition is Based on a Dual-Channel of Expression and Posture

Section Data Mining-based Mental State Recognition of Undergraduates in Medical College introduces the healthcare publicity in medical college, through the mental state recognition signals of undergraduates in medical college to judge whether they have hidden illness. In addition to the mental state, facial expressions can also reflect the emotions of undergraduates. However, it is not enough to take facial expressions as the perception of emotions. In this section, the emotion recognition method combining facial expression and posture is proposed to judge whether undergraduates have hidden illnesses, on the other hand, to achieve the purpose of healthcare publicity. This section uses vision-based expression and posture to expand the channels of emotion recognition and proposes a dual-channel emotion recognition model based on deep learning during healthcare lectures or electives in medical college. The model consists of three parts: data preprocessing, feature extraction, and multimodal fusion. To provide the input streams of the expression channel and posture channel, the raw data are preprocessed first, including face detection and posture video sequence size processing. The CNN has a strong learning ability of image features and does not rely on manual experience. A 3DCNN is specially used for human action recognition, which can learn space-time features at the same time. Therefore, this section adopts two networks to model the facial expression image information, posture appearance, and movement information, respectively. Finally, the output of the dual-channel is weighted and fused and the final classification result is obtained.

A. *Data preprocessing*
  Data preprocessing includes facial expression detection and posture video sequence size processing, and data acquisition is still through brainwave sensors. To provide the input stream of the expression channel, this study uses multi-task CNN (MTCNN) for facial expression detection. All frames are processed through MTCNN to obtain the facial image and adjusted to 96 × 96 pixels. The input of the posture channel in the dual-channel is a video sequence, and the size of all video frames is uniformly adjusted to 510 × 680 pixels.
B. *Feature extraction*
  a) Expression channel
  To obtain facial expression information, the deep separable CNN mini Xception (38) is used for feature extraction. The network model of mini Xception is derived from Xception architecture. The deep separable convolution can make more effective use of model parameters, and the residual connection module can speed up the convergence process. The learning rate in the training stage is set to 0.2 randomly, and the batch size is set to 32 by referring to the Dian et al. (39).
  b) Posture channel
  To obtain posture emotion information, the C3D network is used for feature extraction. Studies have shown that both expression and posture information play important roles in perceiving emotions from physical expression. At the same time, effective space-time information is critical for video sequences. C3D can simply and efficiently learn time and space features, and pay attention to expression and posture information, which is suitable for extracting emotional features of posture. The initial learning rate in the training stage is set to 0.001 by comparison of the training set, and batch size is set to 10 by referring to the Han et al. (40).
C. *Multimodal fusion*
  The expression channel and posture channel have their own advantages in obtaining the characteristic information. Weighted-sum is used for decision level fusion. After learning features using a neural network, the posterior probability of category is obtained after the full connection layer, and the posterior probability of the output of expression and posture channels is on the weighted-sum operation. Since the facial expression is the main channel, the weight of the expression channel and posture channel are set to 0.7 and 0.3, respectively.

## Experiment and Results Analysis

### Setup

In this study, the experiment is performed under the environment of Python-based deep learning framework TensorFlow, and experiments are executed on a computer with an Intel i9-11900 2.5 GHz CPU, 32GB of RAM (3,333 MHz), and NVIDIA RTX 3080 Ti, 12GB GDDR6X GPU. The sample data distribution of the simulation of mental state recognition signals of undergraduates in a medical college is shown in Table 1. Additionally, three methods are selected for mental state recognition comparison on the same test platform, which are M-SVM2 (26), PoPP (27), and MD-IMA (28), and three benchmarks can also be used for the simulation of emotion recognition.

**Table 1:** Sample data distribution of mental state recognition signals of undergraduates in medical college simulation.

**No**.	**The number of training samples**	**The number of test samples**
1	200	100
2	100	50
3	200	100
4	150	75
5	200	100
6	150	75
7	200	100
8	180	90
9	300	150
10	100	50

### Comparison Analysis

#### Mental State Recognition of Undergraduates in Medical College

The simulation results are analyzed by using the accuracy, false match rate, and false non-match rate of mental state recognition signals of undergraduates in medical college. The simulation results of mental state recognition signals of undergraduates in medical college are counted respectively.

**Figure 2:**
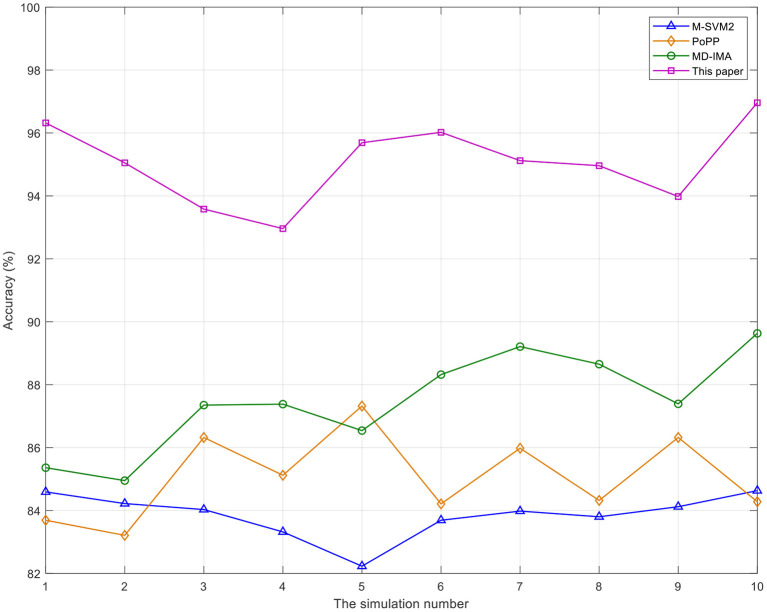
The comparison of the accuracy of mental state recognition of undergraduates in medical college by different methods.

As can be seen from Figure 2, the recognition accuracy of the method proposed in this study is always the highest, while the recognition accuracy of the other three benchmarks is not too high. The recognition accuracy rate can well reflect the recognition of the psychological state of medical college undergraduates. The high recognition accuracy rate of the proposed method can ensure that the mental state of medical college undergraduates in lectures or electives can be well recognized, to improve the ability to judge whether medical college undergraduates hide their illness through their mental state, At the same time, it can clearly find the degree of medical college undergraduates' mastery of healthcare knowledge. With the increasing number of simulation times, the recognition accuracy of the three benchmarks does not exceed 90%. In contrast, the proposed method has been maintained at more than 90%. The recognition accuracy of PoPP varies from high to low and is unstable, the recognition accuracy of MD-IMA shows an upward trend, and the recognition accuracy of M-SVM2 is always low.

**Figure 3:**
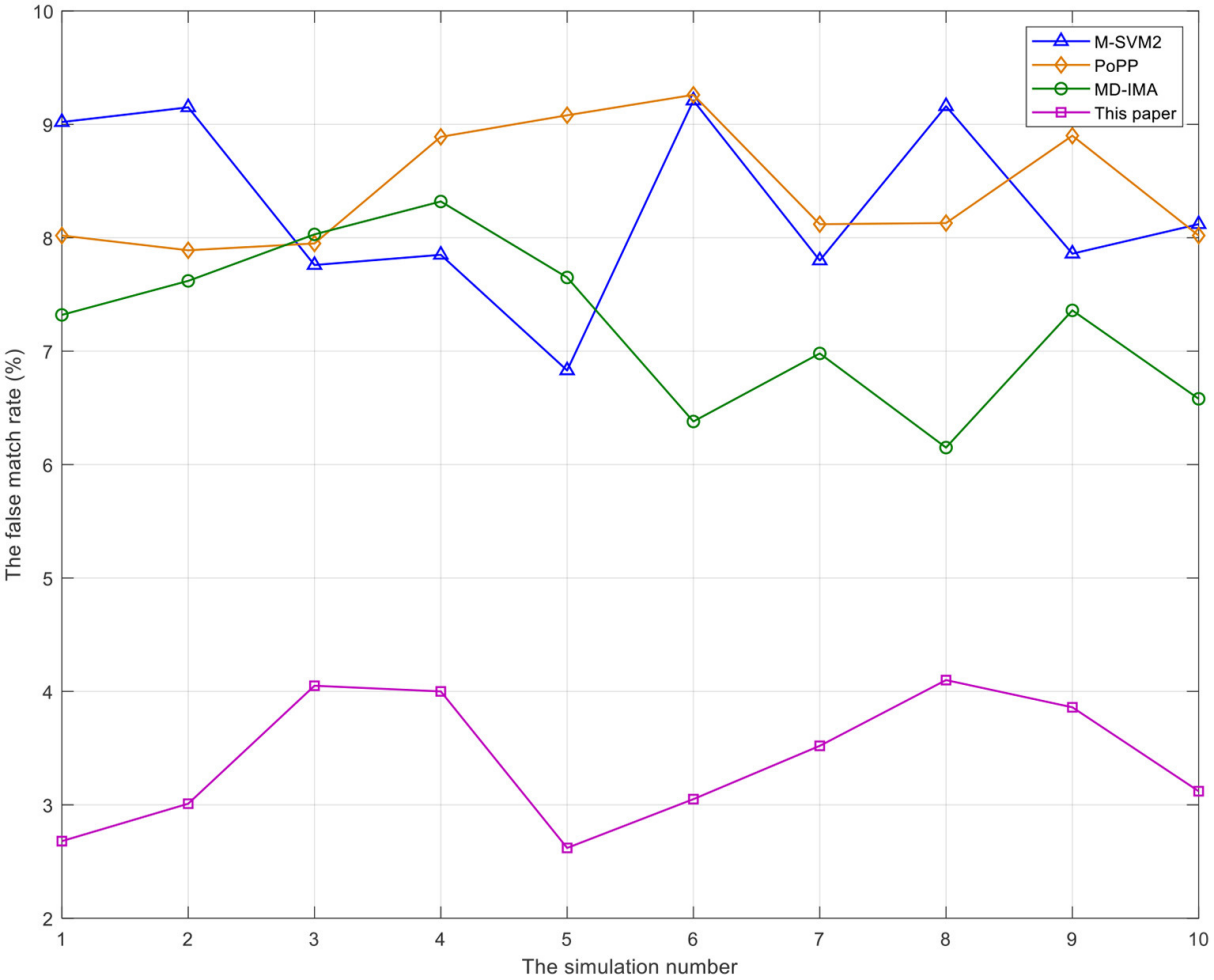
The comparison of false match rate of mental state recognition of undergraduates in medical college by different methods.

**Figure 4:**
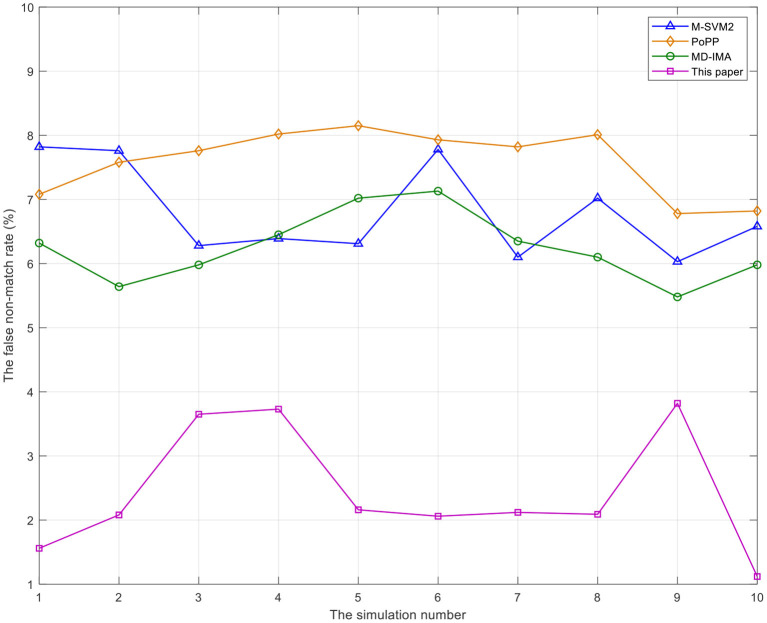
The comparison of false non-match rate of mental state recognition of undergraduates in medical college by different methods.

As can be seen from Figures 3, 4, the false match rate and false non-match rate of the proposed method in this study are always the lowest, almost below 4%. After the 8th simulation, the false match rate of the proposed method shows an obvious downward trend, while the false non-match rate of the proposed method shows a downward trend after the 9th simulation, and the false match rate of the other three benchmarks are all above 5% and not so stable. False match rate and false non-match rate are significant in healthcare lectures or electives. If recognized incorrectly, they are likely to have a negative impact on the mental aspect of medical college undergraduates, further disrupting their desire for healthcare knowledge. The method proposed in this study always keeps low false match rate and false non-match rate, so as to provide a good guarantee for medical college undergraduates in healthcare lectures or electives.

**Figure 5:**
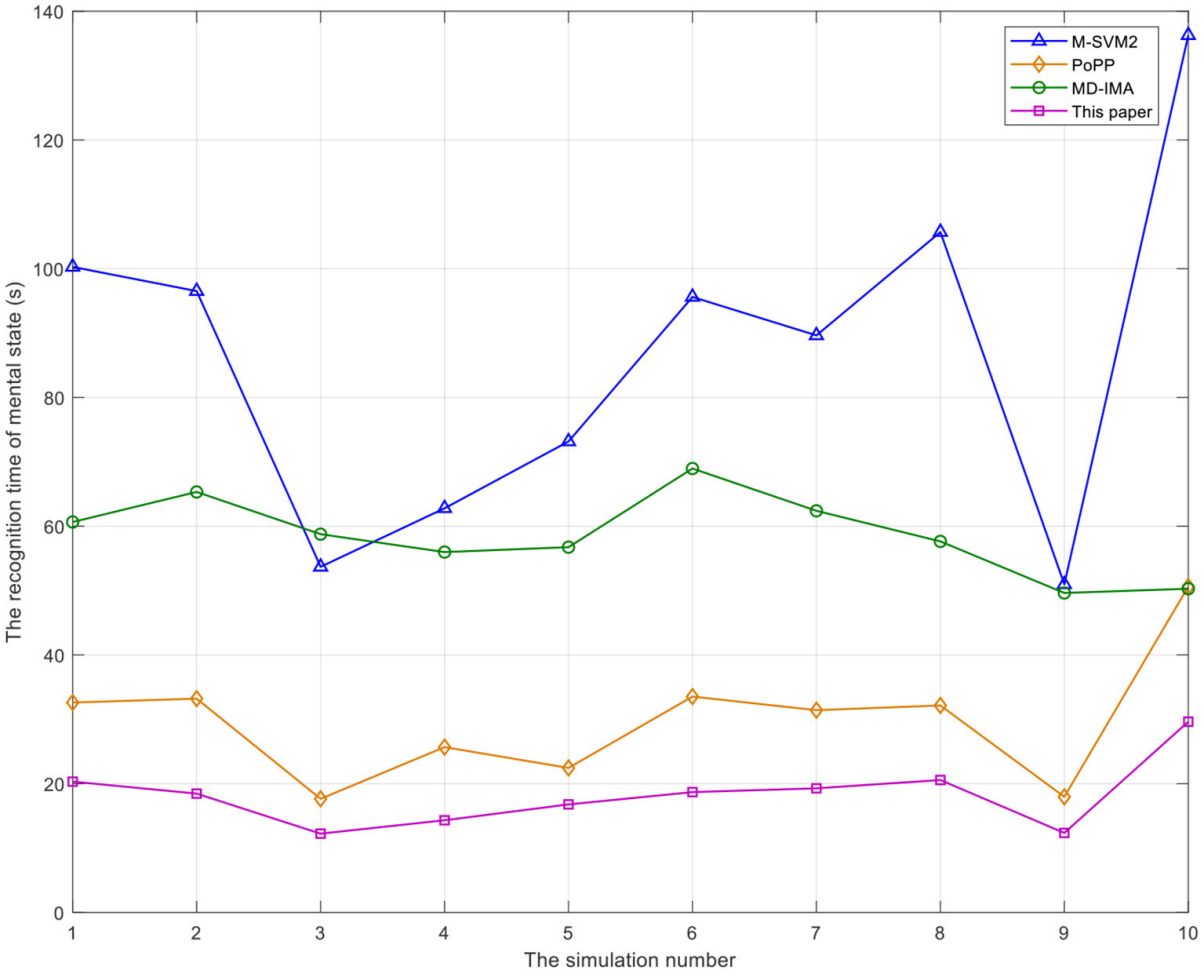
The comparison of recognition time of mental state recognition of undergraduates in medical college by different methods.

As can be seen from Table 2; Figure 5, the recognition time of the proposed method in this study is relatively low. This is because the method proposed in this study uses the ReLU activation function of CNN to make network training faster. Compared with the sigmoid and tanh activation function, the derivative is easier to be obtained. Meanwhile, the ReLU activation function increases the non-linearity of the network and prevents the gradient from disappearing. When the value is too large or too small, the derivative of sigmoid and tanh is close to zero, and ReLU is the unsaturated activation function, there is no such phenomenon. In addition, the ReLU activation function makes the mesh sparse and reduces overfitting. Low recognition times play an important role in healthcare lectures or electives to ensure timely problem detection and facilitate timely decision making. It also can be seen from Table 2 that the recognition time of the proposed method is 78.9% lower than that of M-SVM2.

**Table 2:** Recognition time of mental state recognition of undergraduates in medical college by different methods.

**No**.	**M-SVM2**	**PoPP**	**MD-IMA**	**This paper**
1	100.28	32.61	60.65	20.32
2	96.52	33.21	65.32	18.46
3	53.69	17.65	58.76	12.24
4	62.78	25.68	55.98	14.32
5	73.16	22.45	56.74	16.78
6	95.61	33.53	68.96	18.69
7	89.64	31.42	62.39	19.28
8	105.69	32.15	57.64	20.57
9	50.97	17.96	49.63	12.35
10	136.26	50.62	50.28	29.63
Average	86.46	29.73	58.64	18.26

By comparing the recognition time of undergraduates' mental state recognition, it can be found that the average recognition time of undergraduates' mental state recognition in this study is 18.26 s, while the average modeling time of M-SVM2, PoPP, and MD-IMA are 86.46, 29.73, and 58.64 s, respectively. It can be seen that the method proposed in this study shortens the recognition time of undergraduates' mental state recognition, accelerates the speed of undergraduates' mental state recognition, and has a good effect on the publicity of healthcare.

#### Emotion Recognition

To verify the effect of expression and posture on emotion recognition during healthcare lectures or electives in medical college and the contribution of dual-channel fusion patterns, the simulation on single pattern emotion recognition is conducted. The pre-processing training set is used for data training, and the expression sequence and posture sequence are corresponding to each other. The testing set was used for 10 tests, and the average recognition rate was used as the evaluation index.

**Figure 6:**
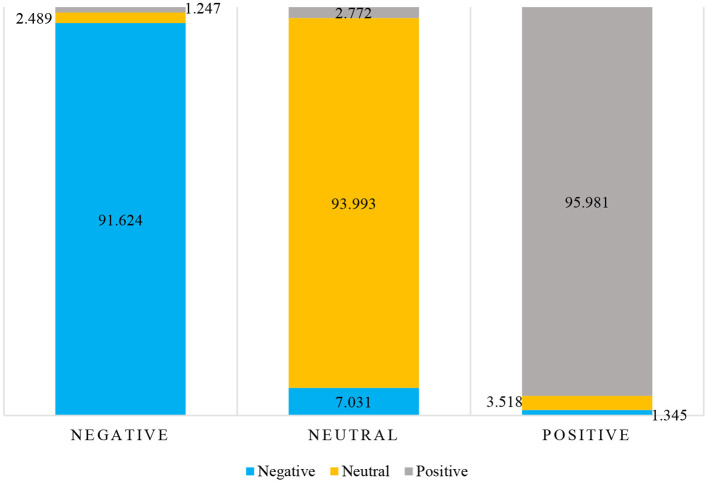
Percentage stacked column chart of facial emotion recognition confusion matrix.

**Figure 7:**
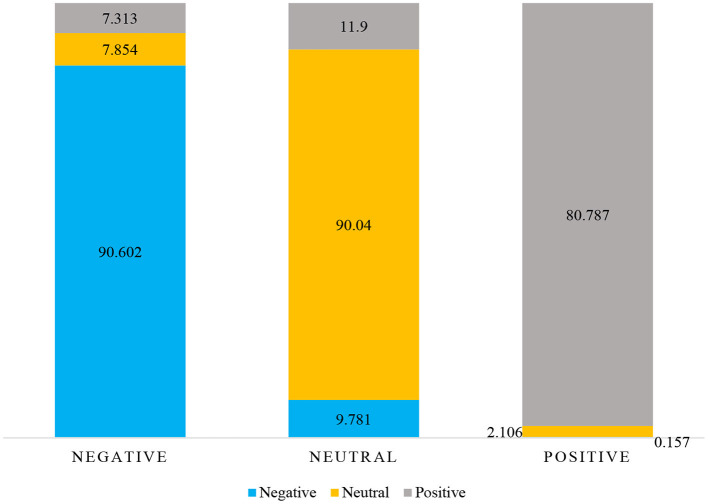
Percentage stacked column chart of posture emotion recognition confusion matrix.

As can be seen from the simulation results in Table 3, the facial expression is of great significance to emotion recognition with an average recognition rate of 94.698%. As can be seen from the confusion matrix in Table 4; Figure 6, the effect of perceiving negative emotion through facial expression is the worst, and it is easy to misjudge as neutral emotion. Posture is diagnostic in emotional expression and can spontaneously reveal some emotional clues with an average recognition rate of 88.024%. As can be seen from the confusion matrix in Table 5; Figure 7, the posture perception of positive emotions is the worst.

**Table 3:** The results of emotion recognition during healthcare lectures or electives in medical college.

	**Expression**	**Posture**	**Dual-channel fusion**
The average recognition rate (%)	93.584	88.062	95.017

**Table 4:** Facial emotion recognition confusion matrix during healthcare lecture or electives in medical college.

**Emotion state**	**Negative**	**Neutral**	**Positive**
Negative	91.624	7.031	1.345
Neutral	2.489	93.993	3.518
Positive	1.247	2.772	95.981

**Table 5:** Posture emotion recognition confusion matrix during healthcare lecture or electives in medical college.

**Emotion state**	**Negative**	**Neutral**	**Positive**
Negative	90.602	9.781	0.157
Neutral	7.854	90.040	2.106
Positive	7.313	11.900	80.787

**Figure 8:**
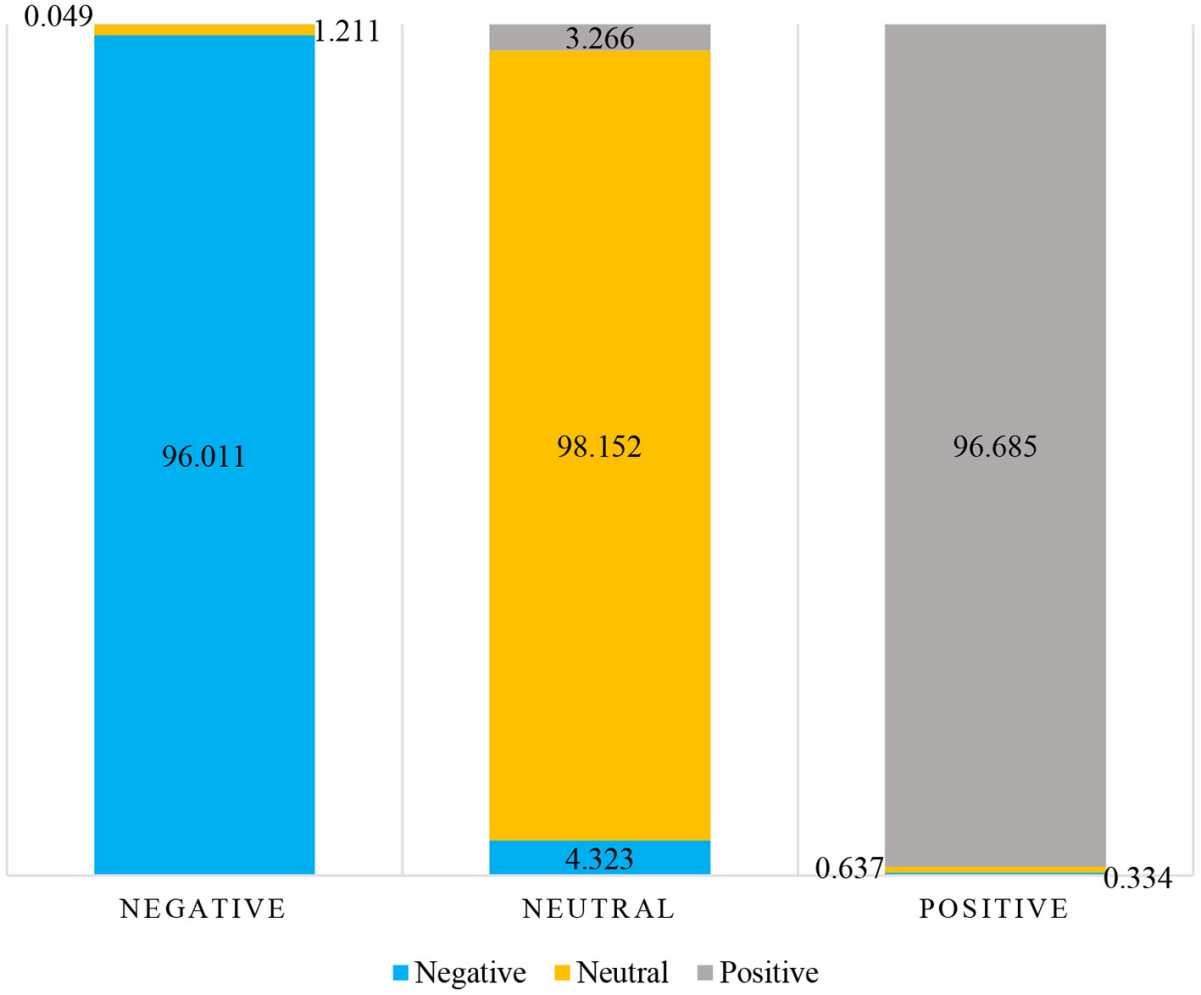
Percentage stacked column chart of dual-channel emotion recognition confusion matrix.

As shown in Table 6; Figure 8, dual-channel fusion emotion recognition verifies the effectiveness of emotion recognition through facial expression and posture, and the average recognition rate is greatly improved, which is higher than the facial emotion recognition and posture emotion recognition separately. By comparing the single pattern and dual-channel fusion emotional confusion matrix can be more intuitive to see the advantage of the dual-channel fusion mode: when expression channel and posture channel are fused, the limitations of expression perception of negative emotion and posture perception of positive emotion are complementarily improved, and the recognition rate of neutral emotion is improved, so as to improve the overall judgment accuracy. It shows that facial expression and posture contribute to emotion recognition, and the expressed information can effectively complement each other. Combining facial expression and posture can improve the ability and reliability of recognizing emotional state during healthcare lectures or electives in medical college.

**Table 6:** Dual-channel emotion recognition confusion matrix during healthcare lecture or electives in medical college.

**Emotion state**	**Negative**	**Neutral**	**Positive**
Negative	96.011	4.323	0.334
Neutral	1.211	98.152	0.637
Positive	0.049	3.266	96.685

## Conclusions

Healthcare has always been neglected in colleges, resulting in negative influence and some unsafe incidents. In the lectures or electives of medical colleges, healthcare should be taken as the publicity strategy to promote the awareness of healthcare among undergraduates through medical undergraduates' better understanding of healthcare. However, in the healthcare lectures or electives, the master's degree of medical undergraduates and whether to hide the illness are the focus of this study. To recognize the mental state of undergraduates in medical college, brainwave sensors are introduced to collect mental state signals of undergraduates in medical college. Then, empirical wavelet transform is introduced to denoise the mental state signals of undergraduates in medical college, and LDA is used to extract their mental state recognition features. So CNN is used to construct the mental state recognition model of undergraduates in medical college. In addition, the dual-channel emotion recognition method combining facial expression and posture is proposed to judge whether undergraduates have hidden illnesses, on the other hand, to achieve the purpose of healthcare publicity. The simulation results reveal that the proposed method can perfectly recognize the mental state and emotion to meet the requirements in lectures or electives.

The research of this study is aimed at the recognition of individual mental states and emotions in public space, and there is group-level in public space, which is also meaningful for the calculation of the overall mental states and emotions of the group-level. The next step is to study multi-scale mental state and emotion recognition in public space. CNN is used for static output, while recurrent neural network (RNN) can be used to describe the output of continuous state in time with memory function. Therefore, the combination of CNN and RNN can be considered for training in the next step.

## Data Availability Statement

The raw data supporting the conclusions of this article will be made available by the authors, without undue reservation.

## Author Contributions

CW contributes to writing and methodology. LZ contributes to data analysis and information research. Both authors contributed to the article and approved the submitted version.

## Conflict of Interest

The authors declare that the research was conducted in the absence of any commercial or financial relationships that could be construed as a potential conflict of interest.

## Publisher's Note

All claims expressed in this article are solely those of the authors and do not necessarily represent those of their affiliated organizations, or those of the publisher, the editors and the reviewers. Any product that may be evaluated in this article, or claim that may be made by its manufacturer, is not guaranteed or endorsed by the publisher.
